# MiR-205 inhibits cell apoptosis by targeting phosphatase and tensin homolog deleted on chromosome ten in endometrial cancer ishikawa cells

**DOI:** 10.1186/1471-2407-14-440

**Published:** 2014-06-14

**Authors:** Guiyu Zhang, Xinxin Hou, Yue Li, Meng Zhao

**Affiliations:** 1 Department of Gynecology, Qilu Hospital, Shandong University, 107 Wenhuaxi Road, Jinan 250012, P.R. China; 2 Department of Gynecology, Weihai Municipal Hospital, 70 Heping Road, Weihai 264200, P.R. China; 3 Department of Gynecology, Zibo Maternal and Child Health Hospital, 11 Xingyuandong Road, Zhangdian District, Zibo 255000, P.R. China

**Keywords:** Endometrial cancer, microRNA, PTEN, AKT pathway

## Abstract

**Background:**

MicroRNAs (miRNAs) are frequently dysregulated in human cancers and can act as either potent oncogenes or tumor suppressor genes. In the present study, we intend to prove that the gene PTEN (phosphatase and tensin homolog deleted on chromosome ten) is a target gene of miR-205 and to investigate the suppressive effects on PTEN transcriptional activity by enhancing miR-205 expression in endometrial cancer Ishikawa cells.

**Methods:**

Using Ishikawa cells as model systems, we up-regulated miR-205 expression by transient transfection with miR-205 mimics. A luciferase reporter assay, qRT-PCR and western blotting assays were used to verify whether PTEN is a direct target of miR-205. Meanwhile, the modulatory role of miR-205 in the AKT (protein kinase B) pathway was evaluated by determining the AKT phosphorylation. As a biological counterpart, we investigated cell apoptosis using flow cytometry.

**Results:**

Our data indicate that miR-205 down-regulates the expression of PTEN through direct interaction with the putative binding site in the 3′-untranslated region (3′-UTR) of PTEN. Moreover, we documented the functional interactions of miR-205 and PTEN, which have a downstream effect on the regulation of the AKT pathway, explaining, at least in part, the inhibitory effects on Ishikawa cell apoptosis of enhancing miR-205 expression.

**Conclusions:**

For the first time, we demonstrate that the expression of PTEN is directly regulated by miR-205 in endometrial cancer cells and leads the inhibition of cellular apoptosis. This relationship could be targeted for new therapeutic strategies for endometrial cancer.

## Background

Endometrial cancer (EC) is one of the most common female pelvic malignancies, and its incidence has recently increased worldwide [1]. While early-stage EC is generally considered to have a good prognosis, the nature of the disease is heterogeneous, and there is a significant group of patients with a high risk of cancer recurrence and death [2,3]. The lack of effective therapy for patients with advanced-stage and recurrent disease is to some extent a reflection of an incomplete understanding of the molecular basis of endometrial carcinogenesis [4]. The identification of effective targets for EC tumorigenesis and treatment would have a major impact on women’s health.

MicroRNAs (miRNAs) are small non-coding RNA transcripts that influence cell function via modulation of the post-transcriptional activity of multiple mRNA gene targets. Gene silencing by miRNAs is primarily achieved by targeting the 3′-untranslated region (3′-UTR) of mRNAs and inducing translational silencing [5]. Recent studies have demonstrated that miRNAs may influence human cancer development and can act as either potent oncogenes or tumor suppressor genes [6]. Some investigators have suggested that miRNA signatures can be considered promising biomarkers for the early detection and prognosis of EC [7]. Although a large number of miRNAs have been identified to date in EC, the role for many of them in tumorigenesis and their underlying mechanisms remain unclear.

Using an miRNA microarray to detect differential expressions of miRNAs in EC tissues, we have identified several miRNAs that are of importance for further research. Of these miRNAs, we focused on miR-205, which was found to be overexpressed in EC [8], a finding that is consistent with other studies [9-11]. Recently, miR-205 has been associated with a variety of tumors. Of interest, miR-205 was expressed in a low level and functioned as a tumor suppressor gene in breast cancer and prostate cancer [12-14]; however, in studies of non-small cell lung cancer, bladder cancer and head and neck squamous cell carcinoma [15], miR-205 was overexpressed and acted as an oncogene. Although many properties of miR-205 have been revealed, its targets and its role in EC remain to be evaluated. Using a target gene prediction system, we proposed that PTEN (phosphatase and tensin homolog deleted on chromosome ten) is a putative target gene of miR-205. PTEN is a tumor suppressor that regulates cell survival and proliferation by antagonizing phosphatidylinositol 3-kinase/protein kinase B (PKB/AKT) signaling [16]. In human EC, reduced expression of PTEN and overexpression of phosphorylated AKT (pAKT) are frequently correlated with tumor progression and a poor prognosis. miR-205 expression has an inverse correlation with the PTEN protein using the non-parametric Spearman correlation analysis [17]. PTEN was predicted to be a target of miR-205 by previous studies [18,19]; however, this prediction has not been validated in EC.

In the present study, we sought to determine whether there are any target relationships between miR-205, the tumor suppressor gene PTEN and their underlying mechanisms in Ishikawa cells. Significantly, we show that miR-205 directly targets PTEN by binding to its 3′-UTR, leading to the inhibition of PTEN translation and the activation of the AKT pathway. We also show that enhanced miR-205 expression inhibited cellular apoptosis in EC cells. Therefore, we suggest that miR-205 is a specific oncogene in EC and a novel target for EC therapy.

## Methods

### Cell lines and culture conditions

Ishikawa cells were obtained from the European Collection of Cell Cultures (ECACC, Wiltshire, UK) and are preserved by the Key Laboratory of Gynecologic Oncology at Qilu Hospital. Cells were cultured in MEM medium supplemented with 5% fetal bovine serum (FBS, Gibco, USA) and were incubated at 37°C in a humidified chamber supplemented with 5% CO2.

### Normal endometria collection

The normal endometria were obtained from 15 patients with benign uterine diseases who underwent surgical hysterectomies at Qilu Hospital, Shandong University. The endometria from these patients were snap-frozen in liquid nitrogen and stored at -80°C immediately after excision. These patients did not receive any treatment prior to surgery. This study was approved by the Research Ethics Board of Qilu Hospital.

### Reporter vectors and constructs

The 2.7 kb 3′-UTR sequence of PTEN was amplified from genomic HEK293 cell DNA and subcloned into the XhoI site of the dual luciferase reporter vector (pmiR-RB-REPORT™, RIBOBIO, China). The mutant construct of PTEN 3′-UTR was generated using a KOD-Plus-Mutagenesis Kit (TOYOBO, Japan) by site-directed mutagenesis via established methods [20].

### Oligonucleotide and cell transfection

MiR-205 mimics, designed to mimic endogenous mature miR-205, were purchased from GenePharma (Shanghai, China) as well as scrambled oligonucleotides, which did not produce identifiable effects on miR-205 function, used as negative control miRNA. Cells were grown to 60% confluence and miR-205 mimics or negative controls were transiently transfected using Lipofectamine 2000 (Invitrogen, USA) according to the manufacturer’s specifications. MiR-205 mimics and plasmid co-transfections were also performed using Lipofectamine 2000. Twenty-four hours after transfection, cells were plated for an apoptosis assay or harvested for the luciferase reporter assay. Cells were harvested for RNA and protein analyses at forty-eight hours after the transfection.

### RNA isolation and quality control

Total RNA was isolated from cells and normal endometria using the TRIzol reagent (Invitrogen, USA) according to the manufacturer’s instructions as previously described [21]. RNA purity and quality were detected by a spectrophotometer and Denaturing Agarose Gel Electrophoresis. The O.D. A260/A280 ratio should be close to 2.0 for pure RNA (ratios between 1.8 and 2.1 were acceptable).

### Quantitative real-time polymerase chain reaction (qRT-PCR)

cDNA was synthesized from 10 ng of total RNA using the M-MLV Reverse Transcription Kit (Invitrogen, USA) with Bulge-loop™ miR-205 qRT-PCR Primer (synthesized by RIBOBIO, Guangzhou, China). The primers for miR-205 were 5′-CTT GTC CTT CAT TCC ACC GGA-3′ (forward) and 5′-TGC CGC CTG AAC TTC ACT CC-3′ (reverse). Amplification reactions were performed using SYBR Premix Ex Taq (Takara, Japan) according to the manufacturer’s protocol and were carried out using the Light Cycler System in triplicates (Roche Diagnostics GmbH, Mannheim, Germany) as previously described [22]. We also carried out positive and negative control reactions on each plate. The melting curve of each PCR product was determined, and the threshold cycle (Ct) data were determined. MiRNA-205 expression was determined by 2^ˉ△△Ct^ measurements and normalized to U6 [23]. For PTEN qRT-PCR analysis, cDNA was synthesized using the PrimeScript RT reagent Kit with gDNA Eraser (Takara, Japan). The total volume of each real-time PCR reaction was 20 μl containing primers and the SYBR green Master Mix (Takara, Japan). The primers for PTEN were 5′-TGT GGT CTG CCA GCT AAA GG-3′ (forward) and 5′-CGG CTG AGG GAA CTC AAA GT-3′ (reverse). β-actin was used as a normalized control, using the following primers: 5′-GCA CCC AGC ACA CAA TGA AG-3′ (forward) and 5′-GCA CCC AGC ACA ATG AAG-3′ (reverse). All of the PCR products were verified by DNA sequencing.

### Western blotting

Cells were lysed in RIPA buffer with protease and phosphatase inhibitors (Beyotime, Beijing, China) at 4°C for 30 min. Protein concentrations were measured with the BCA Protein Assay Kit (Beyotime, Beijing, China). The western blot analysis was carried out as reported previously [24]. Fifty micrograms of total protein was separated by SDS-PAGE on 10% gel and transferred onto PVDF membranes (Millipore, USA) at 200 mA for 1.5 h. Membranes were incubated with primary antibodies overnight at 4°C. Then, membranes were incubated with secondary antibodies for 1 h at room temperature. The immunoreactivity of proteins was detected using ECL Reagent (Millipore, USA). The mean density of each band was quantified using Image J software with β-actin used as an internal control. Specific primary antibodies used in this study were purchased from Bioworld (USA).

### Cell apoptosis assay

The cell apoptosis assay was performed using flow cytometry and was detected with the Annexin V-FITC/PI Apoptosis Detection Kit (BestBio, China). Cells were cultured in 96-well plates and treated with the agents indicated in the figure legends according to the manufacturer’s instructions. Cells were resuspended in 400 μl Annexin V binding buffer and subsequently incubated with 5 μl Annexin V-fluorescein isothiocyanate for 15 minutes in room temperature; then, 10 μl of propidium iodide was added. Experimental data were analyzed using Tree Star FlowJo software (version 8).

### Luciferase assay

Cells were grown to approximately 60% confluence in 24-well plates and co-transfected with pmiR-RB-PTEN-3′-UTR (wild type or mutant) plus miR-7 mimics or negative control miRNA using Lipofectamine 2000. After 24 hours of incubation, firefly and Renilla luciferase activities were evaluated using the Dual-Luciferase Reporter Assay system (Promega, USA) according to the manufacturer’s protocol and reported as relative luciferase units (firefly luciferase/Renilla luciferase).

### Statistical analysis

All results, including transfection, were repeated using independent experiments in triplicate. Comparisons between the two groups were performed with Student’s *t*-test (with or without a Welch correction). When more than two groups were compared, significant differences were determined by a one-way ANOVA or with nonparametric tests for small groups of subjects. Differences in p values of <0.05 were considered statistically significant. All statistical analyses were performed using SPSS 17.0 software.

## Results

### MiR-205 is expressed at higher levels in Ishikawa cells compared with normal endometrial tissues

**Figure 1:**
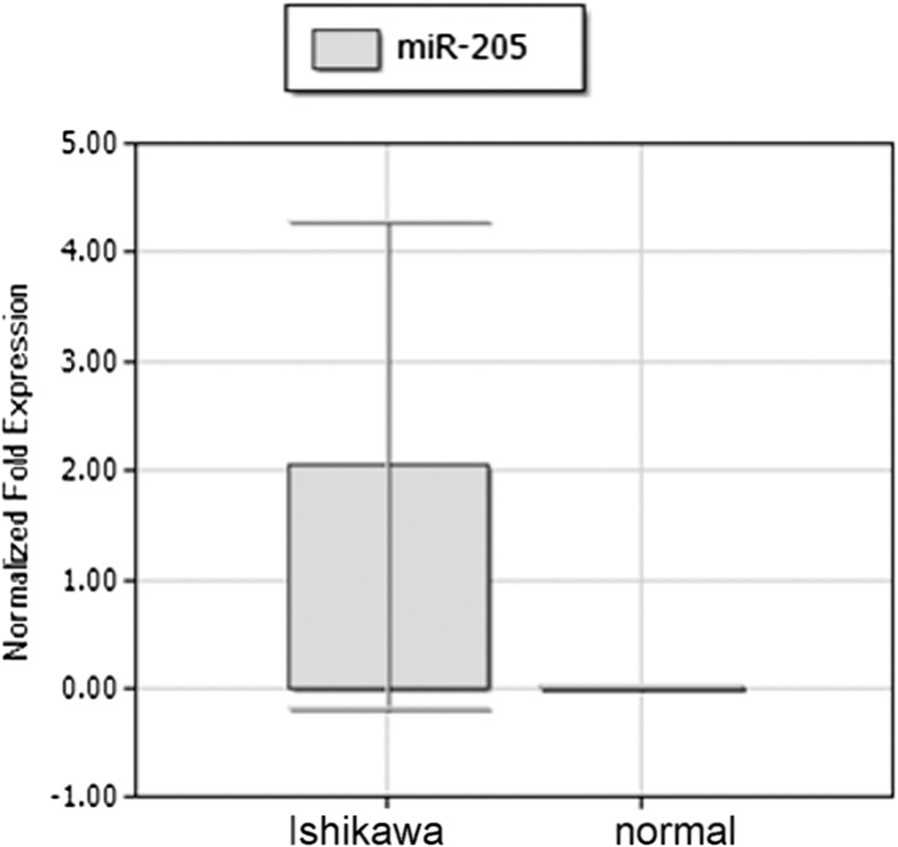
**Expression of miR-205 in the Ishikawa cell line.** qRT-PCR verified that miR-205 was highly expressed in Ishikawa cells compared with normal endometrium (p = 0.002). The results are presented as the means ± SD of three independent experiments.

To analyze whether miR-205 was expressed differently between endometrial cancer cells and normal endometria, we utilized qRT-PCR to detect miR-205 expression levels. As shown in Figure 1, Ishikawa cells had a high mRNA expression. In contrast, low miR-205 expression was detected in normal endometria. The difference in miR-205 expression between Ishikawa cells and normal endometria was statistically significant (95% CI 0.89-2.35, p = 0.002). There are few studies on the role of miR-205 in EC. These results may suggest an oncogenic role for miR-205 in EC.

### MiR-205 directly targets PTEN expression through binding to the 3′-UTR region of PTEN

**Figure 2:**
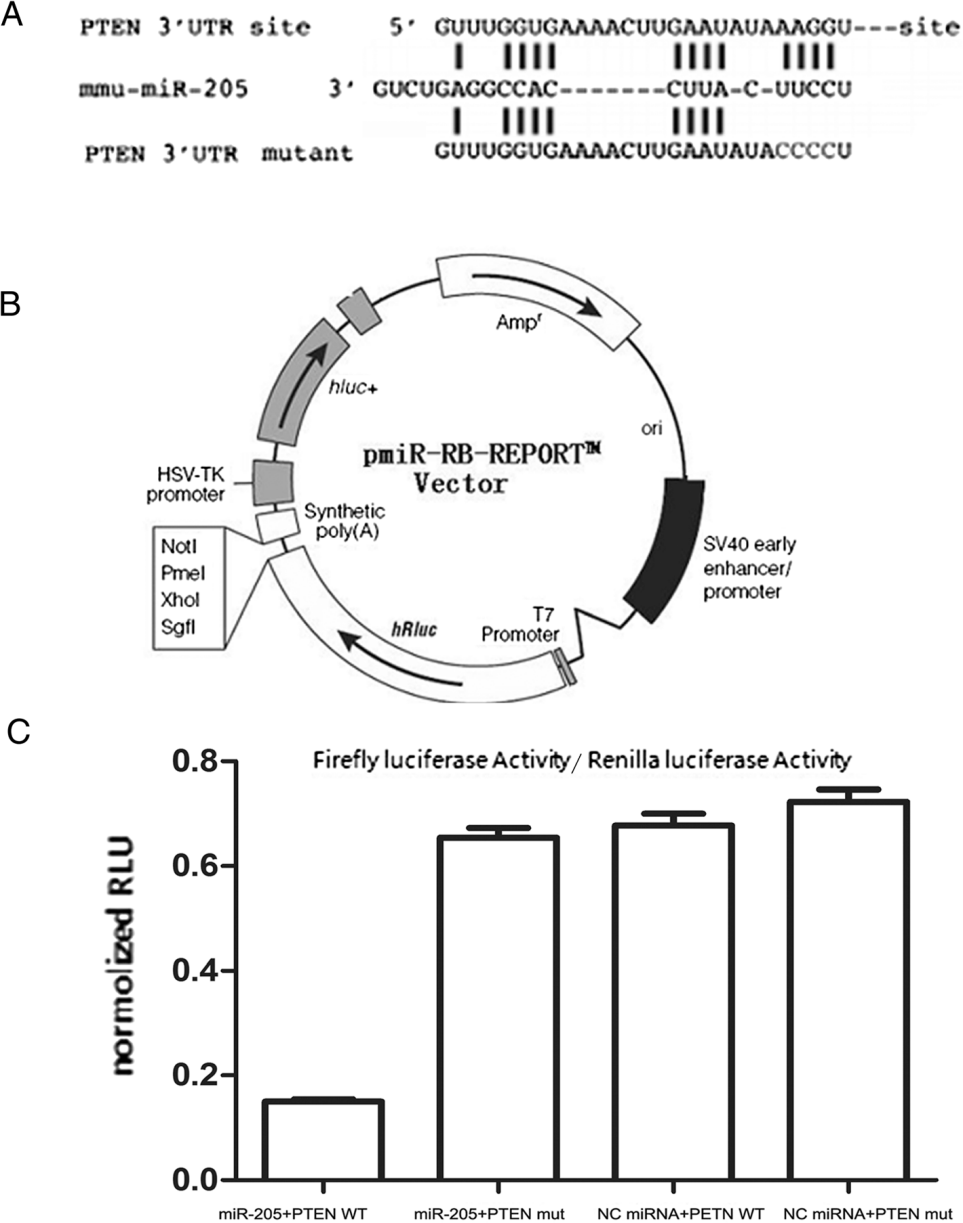
**MiR-205 targets PTEN at 3′-UTR. (A)** Sequences of miR-205 binding sites with PTEN 3′-UTR. Red nucleotides are mutated nucleotides of PTEN 3′-UTR. **(B)** Schematic of the Dual-Luciferase pmiR-RIBOBIO reporter vector constructs. The 3′-UTR region of PTEN was subcloned into the Dual-Luciferase reporter vector. **(C)** Effect of miR-205 on the luciferase activity of Luc-PTEN 3′-UTR WT and Luc-PTEN 3′-UTR-mut. The wild type or mutant vectors were co-transfected with miR-205 mimics or negative control miRNA in Ishikawa cells. Luciferase activity was significantly decreased in miR-205+ PTEN WT group compared with miR-205+ PTEN mut (p = 0.002) or NC miRNA + PTEN WT groups (p = 0.005). Each sample was normalized to Renilla luciferase activity. (RLU, relative luciferase units: firefly luciferase/Renilla luciferase). Data are the geometric means of three independent experiments, performed in duplicates.

To identify downstream targets of miR-205, we searched for putative miR-205 targets using three programs that predict the mRNA targets of a particular miRNA: miRanda [25], TargetScan [26] and PicTar [27]. From several candidates, we selected the tumor suppressor gene PTEN as a putative miR-205 target. The 3′-UTR of human PTEN contains a region (nucleotides 559–585, Figure 2A) that matched to the seed sequence of miR-205. To determine whether PTEN was indeed directly regulated by miR-205, the wild type 3′-UTR of PTEN and the mutant (Figure 2A) were constructed and inserted into the pmiR-RB-REPORT luciferase plasmid (Figure 2B). The wild type or mutant vectors were co-transfected with miR-205 mimics or negative control miRNA in Ishikawa cells. We observed a significantly decreased luciferase activity of the wild type PTEN 3′-UTR compared with the mutant PTEN 3′-UTR (P = 0.004) (Figure 2C).

### miR-205 negatively regulates PTEN expression and leads to activation of the AKT pathway

**Figure 3:**
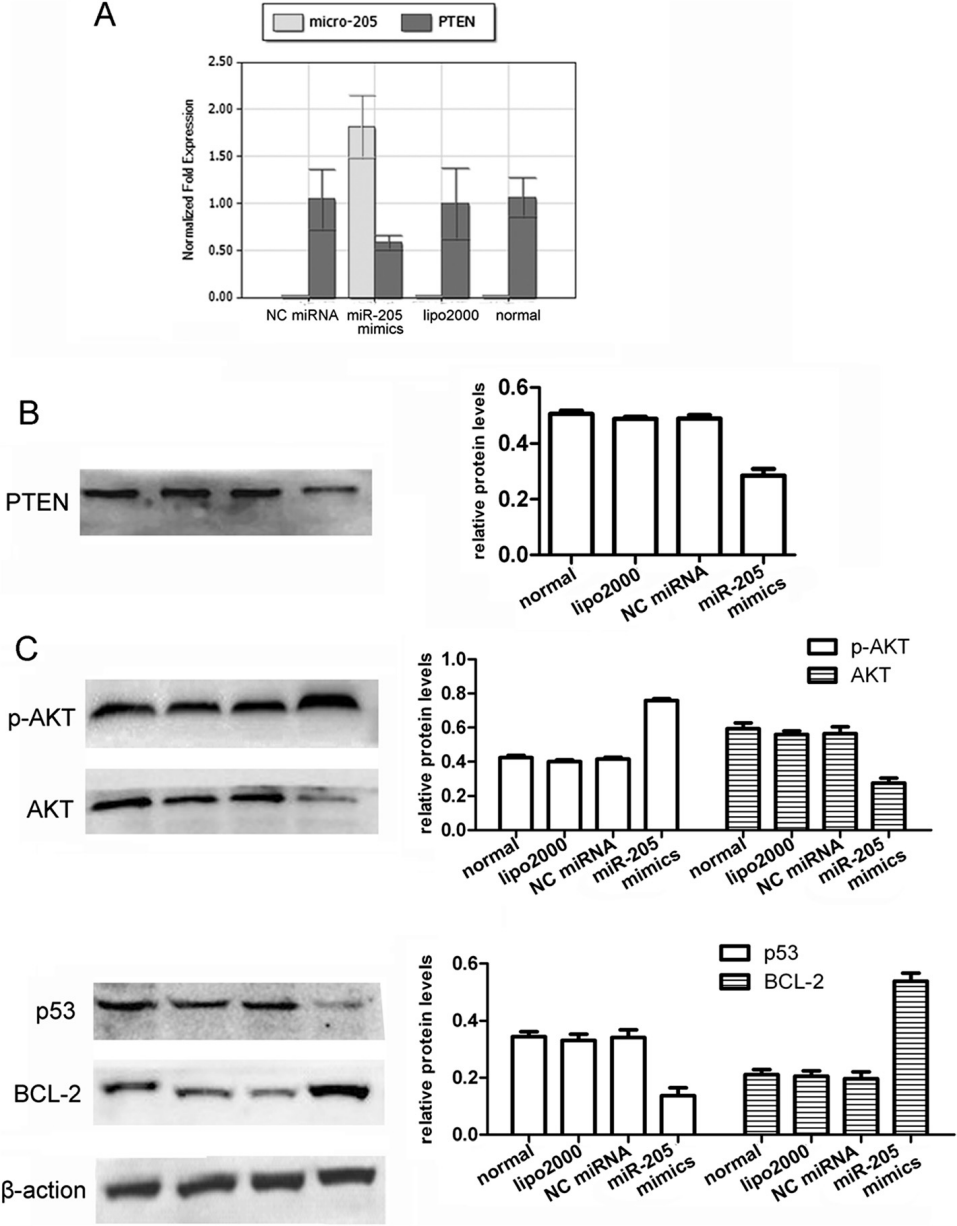
**MiR-205 down-regulates PTEN expression and leads to activation of the AKT pathway. (A)** PTEN mRNA levels were measured by qRT-PCR. Relative PTEN mRNA levels were significantly decreased in miR-205 mimic-transfected cells compared with the NC miRNA group (p = 0.009). **(B, C)** A western blot analysis of PTEN protein levels and its downstream substrate (p-AKT, AKT, p53 and BCL-2) protein levels in Ishikawa cells transfected with miR-205 mimics. The blots were stripped and reprobed with β-actin as a loading control. The enhanced expression of miR-205 reduced PTEN protein levels, and the AKT pathway was significantly regulated compared with the NC miRNA group (p-AKT, p = 0.004, p53, p = 0.02, BCL-2, p = 0.002). The mean intensity of the band was quantified using Image J software. (normal: non-treated cells; lipo2000: cells only treated with lipo2000; NC miRNA: cells transfected with negative control miRNA + lipo2000; miR-205 mimics: cells transfected with miR-205 mimics + lipo2000).

To further assess the correlations between the endogenous levels of miR-205 and PTEN, qRT-PCR and western blot analyses were used to detect the miR-205 and PTEN expression levels in transfected Ishikawa cells. As expected, PTEN mRNA levels were significantly decreased in miR-205 mimic-transfected cells (Figure 3A). In addition, the western blot analysis indicated that the enhanced expression of miR-205 reduced the PTEN protein expression (Figure 3B). Furthermore, the levels of phosphorylated AKT, which is a critical target of PTEN, were elevated by the enhanced expression of miR-205 (Figure 3C). Additionally, the levels of the potential functional downstream substrates p53 and BCL-2 (B cell leukemia-2) were dramatically changed. The reduction in PTEN protein levels was correlated with reduced p53 and increased BCL-2 levels (Figure 3C). These results indicated that miR-205 expression is inversely correlated with PTEN expression and leads to the activation of the AKT pathway in EC cells. Previous studies exploring the AKT regulation of BCL-2 have shown that the AKT kinase can activate the BCL-2 promoter region, as shown by reporter assays [28]. Thus, the expression levels of p53 and BCL-2 were mediated by the activated AKT kinase.

### MiR-205 inhibits EC cell apoptosis in vitro

**Figure 4:**
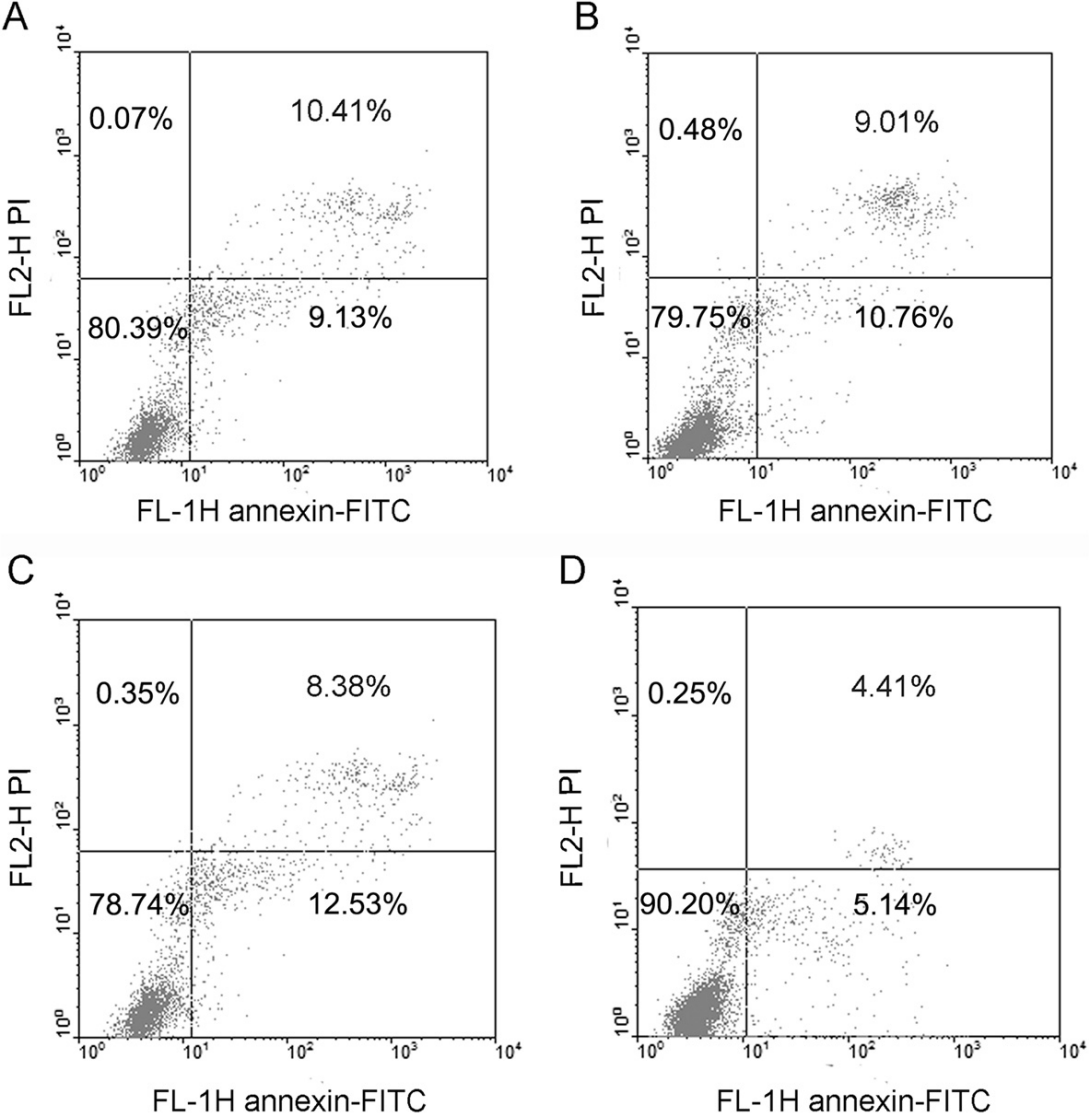
**MiR-205 inhibits Ishikawa cell apoptosis in vitro.** Cell apoptosis rates of Ishikawa cells transfected with miR-25 mimics were significantly decreased compared with the NC miRNA group (p = 0.002). Representative images of the FCM assay of three independent experiments. **(A)** Non-treated Ishikawa cells. **(B)** Ishikawa Cells treated with lipo2000. **(C)** Cells transfected with negative control miRNA + lipo2000. **(D)** Cells transfected with miR-205 mimics + lipo2000.

Given that miR-205 may function as an oncogene in EC, we considered whether miR-205 might have an important role in EC cell apoptosis. The flow cytometry assay demonstrated that the apoptosis rates of Ishikawa cells transfected with miR-205 mimics were significantly decreased compared with those of negative control miRNA-transfected cells (Figure 4). As AKT and BCL-2 are critical for cell survival and proliferation, we considered that miR-205 reduced cell apoptosis partly through activation of the AKT pathway. Taken together, these results indicated that miR-205 inhibits the cellular apoptosis of endometrial cancer cells.

## Discussion

MiRNAs are increasingly implicated in regulating the malignant progression of cancer by directly targeting oncogenes and tumor suppressor genes [29]. Each miRNA can potentially interact with several mRNA targets via base pairing in the 3′-UTR portion. A number of target prediction algorithms, including TargetScan, PicTar and miRanda, relying on seed sequence pairing rules and conservational analysis, have been developed to score possible recognition sites and identify putative gene targets. However, these predictions usually yield a large number of false-positive candidates, and experimental validation is, thus, strictly required [30]. The expression of miR-205 in cancer is controversial because reports have indicated that it is up-regulated or down-regulated in different tumor tissues compared with normal tissues. In the present study, we observed that miR-205 was markedly up-regulated in the Ishikawa cell line compared with normal endometrium. Moreover, several studies using endometrial cancer tissues demonstrated the same results. These findings indicated that enhanced miR-205 expression in EC cells may be important for EC progression.

Many studies have demonstrated that PTEN is mutated or deleted in a great number of human tumors, including EC. Additionally, it is known that PTEN codifies a dual specificity phosphatase, and it is well-known to be required for the phosphorylation and activation of the proto-oncogene AKT [31]. As PTEN is a putative target for miR-205, we next located potential binding sites of miR-205 in the PTEN 3′-UTR region (Figure 2A). A luciferase reporter assay revealed that miR-205 directly interacted with the PTEN 3′-UTR (Figure 2C), and the overexpression of miR-205 diminished PTEN mRNA and protein levels in Ishikawa cells. In addition, we described herein that miR-205 blocks PTEN translation and results in the activation of the AKT pathway (Figure 3). It has been well documented that the constitutive activation of AKT contributes to tumor progression, and regulates several downstream targets (e.g., p53 and BCL-2). Our results are consistent with previous studies that showed decreased p53 protein levels and increased BCL-2 protein levels after up-regulating miR-205 expression. As the p53 and BCL-2 genes are involved in cell growth, apoptosis and proliferation, these results provide the basis for further investigation on the roles of miR-205 in EC cells. Here, we found that the cell apoptosis rate was inhibited by miR-205 (Figure 4), which may promote endometrial cancer development. These results indicated that miR-205 acts as an oncogene and suppresses cellular apoptosis in EC by targeting the PTEN/AKT pathway.

## Conclusion

Our study not only demonstrated the inhibition of cell apoptosis by miR-205 but also provided a possible downstream pathway (the PTEN/AKT pathway) regulated by miR-205 that triggers endometrial cancer progression. Therapeutic targeting of this dysregulated miR-205 may provide a novel treatment strategy for the disease.

## Abbreviations

EC: Endometrial cancer; miRNA: MicroRNA; 3′-UTR: 3′-untranslated region; PTEN: Phosphatase and tensin homolog deleted on chromosome ten; AKT: Protein kinase B; qRT-PCR: Quantitative real-time polymerase chain reaction; BCL-2: B cell leukemia-2.

## Competing interests

The authors declare that they have no competing interests.

## Authors’ contributions

GZ conceived and supervised the study. XH, YL, and MZ performed the experiments. YL and MZ analyzed the data. XH and YL drafted the manuscript, and GZ revised the manuscript. All authors read and approved the final manuscript.

## Pre-publication history

The pre-publication history for this paper can be accessed here:

http://www.biomedcentral.com/1471-2407/14/440/prepub
